# Patterns of Exposure to Multiple Metals and Associations with Neurodevelopment of Preschool Children from Montevideo, Uruguay

**DOI:** 10.1155/2015/493471

**Published:** 2015-01-28

**Authors:** Katarzyna Kordas, Graciela Ardoino, Donna L. Coffman, Elena I. Queirolo, Daniela Ciccariello, Nelly Mañay, Adrienne S. Ettinger

**Affiliations:** ^1^Department of Nutritional Sciences, Pennsylvania State University, University Park, PA 16802, USA; ^2^School of Social and Community Medicine, University of Bristol, Bristol BS8 2BN, UK; ^3^Faculty of Psychology, Catholic University of Uruguay, 16000 Montevideo, Uruguay; ^4^The Methodology Center, Pennsylvania State University, University Park, PA 16802, USA; ^5^Center for Research, Catholic University of Uruguay, 16000 Montevideo, Uruguay; ^6^Faculty of Chemistry, University of the Republic of Uruguay, 18000 Montevideo, Uruguay; ^7^Center for Perinatal, Pediatric, and Environmental Epidemiology, Yale Schools of Public Health and Medicine, New Haven, CT 06510, USA

## Abstract

While it is known that toxic metals contribute individually to child cognitive and behavioral deficits, we still know little about the effects of exposure to multiple metals, particularly when exposures are low. We studied the association between children's blood lead and hair arsenic, cadmium, and manganese and their performance on the Bayley Scales of Infant Development III. Ninety-two preschool children (age 13–42 months) from Montevideo, Uruguay, provided a hair sample and 78 had a blood lead level (BLL) measurement. Using latent class analysis (LCA), we identified four groups of exposure based on metal concentrations: (1) low metals, (2) low-to-moderate metals, (3) high lead and cadmium, and (4) high metals. Using the four-group exposure variable as the main predictor, and fitting raw scores on the cognitive, receptive vocabulary, and expressive vocabulary scales as dependent variables, both complete-case and multiple imputation (MI) analyses were conducted. We found no association between multiple-metal exposures and neurodevelopment in covariate-adjusted models. This study demonstrates the use of LCA together with MI to determine patterns of exposure to multiple toxic metals and relate these to child neurodevelopment. However, because the overall study population was small, other studies with larger sample sizes are needed to investigate these associations.

## 1. Introduction

Worldwide, millions of children are exposed to toxic substances and it is accepted that simultaneous exposure to multiple pollutants is the norm rather than an anomaly [1, 2]. Environmental exposure to multiple toxic metals has been reported in preschool [3] and school age children [4–6]. We know that, individually, these metals (lead, cadmium, arsenic, and manganese, among others) contribute to cognitive [7–13] and behavioral deficits in children, including hyperactivity, ADHD, antisocial behavior, and delinquency [14–17]. Yet, surprisingly, we know little about the combined effects of exposure to multiple metals on child cognition and behavior.

Based on animal studies, adverse interactive effects of metals on neurodevelopmental functioning in humans are likely. Several animal studies have shown interactions among toxic metals on the accumulation of metals in different brain regions [18] and on neurotransmitter metabolism [19, 20]. In one study, when lead and manganese were administered together, motor activity and associative (avoidance) learning was reduced [21]. Arsenic and lead given together resulted in higher lead levels in the medulla, pons, and striatum compared to animals receiving lead only [20]. Also, the administration of mining waste, consisting predominantly of arsenic and manganese, but also lead, was associated with lower levels of DOPAC and homovanillic acid in exposed versus control rats [22].

Very few studies have examined the potential synergistic (or antagonistic) effects of multiple metals on children's neurodevelopmental outcomes. There are also differences in how studies accounted for multiple exposures. Some examined the effects of one metal at a time [23–26], some investigated main effects of pairs [27] or groups of metals [8, 28], and only a handful has examined statistical interactions, mostly among pairs of metals [27, 29–33]. Of the studies that have simultaneously measured at least two metals in children, some show evidence of additive or multiplicative effects on cognition or behavior [27, 30–32, 34], but others report no such findings [33, 35]. As a result, we still have little understanding of the extent to which multiple metals interact to produce additive or multiplicative effects on child development.

Another aspect of environmental exposure in young children that has not received much attention is the effect of multiple metals on child outcomes when exposure levels are low. This is important because the vast majority of children worldwide experience exposures that produce low body burdens of metals. Blood lead levels not previously considered to be of concern in children are clearly associated with lower IQ, poorer cognitive performance, and behavior problems [9, 12, 36, 37]. There is much less evidence on other toxic metals but it is likely that they would behave in similar ways due to similar mechanisms of action. It is also possible that low-level exposure to a single metal produces little effect on neurocognitive or behavioral outcomes but has noticeably more damaging effects when cooccurring with other toxicants [30]. Infants and preschool children in Uruguay are exposed to multiple toxic metals at levels that, in other settings, have been associated with cognitive deficits [3]. We have previously examined the association between manganese exposure and neurodevelopment of preschool children while taking into account their hair lead levels [38]. The objective of the present study was to determine the extent to which children are exposed to multiple metals (manganese, lead, cadmium, and arsenic) and how these patterns of exposure are related to measures of neurodevelopment in Uruguayan preschoolers, specifically focusing on the lower spectrum of exposure to these metals. We applied the latent class analysis (LCA) approach to classify children according to the most salient patterns of metal exposure in this population.

## 2. Methods

### 2.1. Study Setting

The study was conducted in Montevideo, the capital of Uruguay, which is a densely populated and industrial coastal city with approximately 1.7 million inhabitants. Previous studies have documented lead and multiple metal exposures in children and adults [3, 39–41]. Anemia in young children is also a concern [42] and cooccurrence of anemia and elevated blood lead levels in Montevideo children has been reported [41]. Exposure to lead may occur from a variety of sources. Leaded gasoline was phased out in 2004 and while blood lead levels have dropped, exposure continues to be a problem [43]. In addition, both formal and cottage industries (cable burning, battery recycling, and scrap-metal recycling) have been shown to contribute to lead exposures in this population [39]. The sources of exposure to other metals have not been clearly identified.

### 2.2. Participant Recruitment

Study recruitment has been described in detail elsewhere [44]. In brief, participants were recruited from among mother-child pairs who had participated in an earlier screening of blood lead levels and anemia in Montevideo [41] and from five preschools located in the study area and enrolling children between 12 and 36 months of age. The preschool directors were contacted by telephone and if they expressed an interest in the study, an in-person meeting was arranged to explain the study purpose and procedure. The directors were asked for permission to send informational letters to parents of the preschoolers. Interested parents contacted the research team and set-up study appointments.

Overall, 109 children between the ages of 13 and 55 months were included in the study sample. Of those, 95 children were aged less than 42 months and therefore eligible for developmental assessment using the Bayley Scales of Infant Development. Ninety-two children (97%) provided a hair sample for the analysis of metal concentrations, 78 (82%) had a blood lead measurement, and 71 (75%) were tested with the Bayley Scales.

### 2.3. Ethical Considerations

This study was approved by the Ethics Committee at the Catholic University of Uruguay and the Office of Research Protections at the Pennsylvania State University. Informed consent was conducted by the coordinating pediatrician or psychologist in Spanish, when potential participants arrived for the first study visit. The consent form was read slowly with pauses for parents to ask questions and for the coordinator to explain procedures in more detail. Parents signed the form and retained a copy for their records.

### 2.4. Procedure

Children and their caregivers were invited to attend two separate evaluation sessions at the Catholic University Faculty of Psychology Practicum Center. One visit focused on the collection of biological samples, anthropometric measurements, and demographic questionnaires. In another visit, maternal IQ, depression, and stress as well as child development were evaluated. If necessary, a third visit was scheduled to finish the assessments that could not be completed in the first two visits. Each visit at the Center lasted 1.5–2 hours. The two visits were scheduled approximately 1 week apart to accommodate other study activities. At the end of the procedure, each child received an age-appropriate toy gift for participating in the study. Caregivers were compensated for their own and the child's bus fare. Children also received a light snack after the blood draw.

### 2.5. Assessments

#### 2.5.1. Blood Lead Concentrations

A phlebotomy nurse collected a nonfasting venous blood sample from the child using a 25-gauge blood collection set with butterfly needle (Vacutainer, Becton Dickinson, Franklin Lakes, NJ). Blood was collected into two tubes: a lithium heparin tube for blood lead determination (Vacutest Plast, Italy) and a serum tube with clot activator and separator gel (Vacutest Plast). A drop of blood was taken immediately after the blood draw from the serum tube for the analysis of hemoglobin using the HemoCue 201+ portable hemoglobinometer (HemoCue, Lake Forest, CA). The instrument was calibrated daily using standard controls (low, medium, and high). The whole blood tube was stored on ice until being transported to the University of the Republic, where it was stored at −20°C until analysis. Blood lead analyses were performed as described previously [44] at “CEQUIMTOX” (Specialized Center for Chemical Toxicology), Department of Toxicology and Environmental Hygiene, Faculty of Chemistry, the University of the Republic of Uruguay. Atomic Absorption Spectrometry (AAS, VARIAN SpectrAA-55B) was used with either flame (detection limit = 2.5 *μ*g/dL) or graphite (detection limit = 2.0 *μ*g/dL) furnace ionization, depending on whole blood volume available. The graphite furnace AAS was used to analyze 38% of blood samples for which volume was low (below 2 mL). Analytical conditions were validated with standard quality assurance and control procedures [45]. For laboratory certification, accuracy is tested with a monthly participation in the Interlaboratory Program for Quality Control for Lead in Blood, Spain (PICC-PbS, 2001). For 95.1% of the blood samples, analytic results were CV < 2%. CEQUIMTOX is enrolled in the CDC's Lead and Multi-Element Proficiency Program.

#### 2.5.2. Hair Metal Concentrations

Hair samples were obtained from children using stainless steel, blunt-tip scissors. Samples were taken from the occipital region, cut close to the scalp, and deposited in clean, white envelopes. An equivalent of the thickness of a pencil was collected (in children this was equivalent to 2.8 ± 0.1 g; range 2.6–3.4 g). For girls with long hair, the strands were isolated, twisted, and cut. For boys and children with short or very fine hair, samples were taken from several areas of the head and collected directly into the envelope. Samples were stored at room temperature until analysis.

The analytical procedure for the determination of metal concentrations in hair has been described previously [38]. Very briefly, samples were washed with 10 mL 1% Triton X-100 solution, after which they were sonicated and rinsed 3 times with double distilled water. After drying at 60°C for 24 hours, the samples were digested in one mL of concentrated HNO_3_ using a high-temperature oven. Subsequently, 20 mL double distilled water was added to dissolve the sample. Samples were analyzed at the Materials Characterization Laboratory at the Pennsylvania State University using Inductively Coupled Plasma Mass Spectrometry (ICP-MS) with Collision Cell Technology (Thermo Fisher Scientific Waltham, MA). The NIST1643 trace element in water solution served as the standard reference material. The instrument detection limit is 0.02, 0.02, and 0.005 *μ*g/L for manganese, arsenic, and cadmium, respectively. All the readings obtained from the ICP-MS are adjusted through division by the actual weight of the hair sample used in analysis and reported as ppm (ng/g). There were no values below the limit of detection (LOD) for hair manganese or hair cadmium. Four values were below the reporting limit for hair arsenic; we used the actual instrument values in the analysis.

#### 2.5.3. Cognitive/Psychological Assessments

All cognitive assessments were conducted by trained psychologists or advanced psychology students supervised by trained psychologists. The testing procedures and tools used in the study have been described previously in detail [46]. Tests and questionnaires were administered to the mother/child individually by experienced researchers (except when the child refused to be alone or the mother did not feel comfortable leaving during testing, which depended mainly on the child's age and comfort level with the parent's absence).

*Wechsler Adult Intelligence Scale III* (WAIS III, TEA Ediciones, S.A., Madrid, Spain) was used to assess maternal IQ. Five subtests were administered, including Similarities, Arithmetic, Vocabulary, Block Design, and object assembly. Scores on these subtests were used to calculate an estimated IQ based on the methodology of López and colleagues [47].

Maternal depressive symptoms were assessed using the Argentinian adaptation of the* Beck Depression Inventory II* (BDI II, Ediciones Paidós, Buenos Aires, Argentina). The BDI II evaluates symptoms in adults corresponding to the depressive disorders in the Diagnostic and Statistical Manual of Mental Disorders IV (DSM IV). The instrument consists of 21 items assessing the components of depression, such as sadness, pessimism, failure, feelings of guilt, self-criticism, and suicidal thoughts. Answers to the questions are scored on a scale value of 0 to 3; higher total scores indicate more severe depressive symptoms (scores greater than 19 are indicative of moderate-to-severe depression).

*Bayley Scales of Infant and Toddler Development, 3rd Edition* (Bayley III, Harcourt Assessment, San Antonio, TX) was used to assess the development of participating children less than 42 months of age. The Bayley III is a validated instrument that assesses several domains of child development. In this study, Cognitive and Language (including receptive and expressive communication) abilities were considered. Bayley III was administered in Spanish in an isolated room, with only the tester and child (alone or with parent) present. The child sat independently in a chair without the caregiver present or was seated on the caregiver's lap (with the caregiver asked not to aid or participate in the testing). The scales of interest were administered in one session unless the child became visibly fatigued, fussy, or uncooperative, in which case the testing was rescheduled.

An abbreviated version of the* Home Observation for Measurement of the Environment (HOME) Inventory* was used to assess children's home environments. The abbreviated version was based on the interview portion of the original HOME Inventory by Caldwell and Bradley and was adapted from the instruments used in the New York Longitudinal Study, as described previously [44]. The questionnaire was translated into Spanish by the study team. Separate questions were used for children younger than 3 years of age and children between 3 and 6 years of age. Among other things, the questions assessed the number of books and toys the child had, how often parents read to the child, how many times per week the child left the house to go to the supermarket and other outings, and how often the child spent time/ate meals with both biological parents.

#### 2.5.4. Questionnaires

During the “clinic” visit, caregivers were asked to respond to a series of questionnaires about the household and the child. Specifically, they were queried on parents' age, education and occupation, family size, size and characteristics of their house, and household possessions. The study nurse read each question aloud to the caregiver and recorded her/his responses.

### 2.6. Statistical Analysis

Summary statistics for participant characteristics were calculated. Children were assigned to latent clusters according to their metal exposure characteristics using specialized software (Latent Gold 4.5, Statistical Innovations Inc., Belmont MA). A latent class or latent cluster is a variable indicating underlying subgroups of individuals based on the measured (observed) characteristics. Although latent class analysis allows missing data on the latent class indicators (i.e., metals), it does not allow missing data on covariates. Thus, we used multiple imputation (MI) via chained equations to impute 25 complete data sets, as described below.

Recent research has found that not including the cluster membership variable in the imputation model can attenuate the relationships between the covariates and cluster membership [48]. One approach for addressing this issue, the two-stage imputation [49], was developed for categorical latent class indicators, whereas our indicators were continuous. We followed a four-step procedure that was analogous to that proposed by Harel et al. [49], as outlined below.

In Step 1, we determined the number of latent clusters by fitting the model to all children who had at least one observed metal indicator. This resulted in only 3 children being removed from the data set (i.e., *n* = 92). We determined the number of clusters based on the Akaike Information Criterion (AIC) and the Bayesian Information Criterion (BIC). We also specified the indicators to be continuous and censored at zero because metal levels are not negative. Based on the chosen latent cluster model, we obtained the posterior predicted probabilities of membership in each latent cluster and assigned each child to the one with the highest probability.

In Step 2, we imputed 25 complete data sets in which the covariates, the outcomes, the measured metal indicators, and the cluster membership variable from Step 1 were all included in the imputation model.

In Step 3, we deleted the cluster membership variable from Step 1 and refit the model in each of the 25 imputed data sets to estimate a new set of posterior predicted probabilities by including the covariates in the model. We did this because Bray and colleagues [48] have shown that when covariates and/or outcomes of cluster membership are of interest, then classifying individuals on the basis of the maximum posterior probability estimated from a model without the covariates (i.e., the model in Step 1) results in an attenuation of effect estimates. Thus, we estimated the cluster membership based only on the indicators (i.e., Step 1) for use in the imputation model (i.e., Step 2) and then reestimated the cluster membership based on the indicators and covariates once we had complete data on the covariates.

In Step 4, following the assignment of children to a latent cluster, we fit regression models on the 25 imputed data sets and combined the estimates across imputations using standard rules [50], which take into account the uncertainty in imputing the missing data.

Unadjusted and covariate-adjusted ordinary least squares regression models were fitted to test for the association between children's multiple exposure to metals and their neurodevelopmental scores. A single exposure variable obtained from latent class analysis was entered as the independent predictor variable. Children's performance on the Bayley III Cognitive, Expressive Vocabulary and Receptive Vocabulary scales were entered as dependent variables into separate regression models. Because the Bayley III has not been standardized in a normative sample of Uruguayan children, only direct/raw scores were used. Covariates used in the multivariate models were chosen based on previous literature, biological plausibility, and previous research in this population, and included child's age and hemoglobin level, maternal IQ and depressive symptoms score, household density, HOME score, and socioeconomic status. We also included a dummy variable representing the tester who administered the Bayley III. Both complete-case analyses (based on the inclusion of children with a complete set of information on all outcomes, predictors and covariates) and regressions based on multiply-imputed data were conducted. Regression analyses were conducted with STATA 12.0 (STATA Corp., College Station, TX).

## 3. Results

The mean age of study children was 29.1 ± 8.3 months and most had healthy hemoglobin values (Table 1). Most children lived in two-parent households that were not affluent but also not overcrowded. The children's mothers were aged 16–43 years and had a mean 9 years of education, fairly low IQ, and a moderate prevalence of depression.

The latent class analysis identified four latent clusters of children according to their levels of the four metals: arsenic, cadmium, lead, and manganese. The values for the 3 and 4 class cluster models are given in Table 2; the 5-class model was not identified. In general, the metals exposure levels were low and it is in this context that the clusters are described and named (Table 3). The first cluster, “low metal exposure,” represented 72% of the children and was characterized by low mean concentrations of hair arsenic, cadmium, and manganese. The second cluster, “low-to-moderate metal exposure,” represented 19% of the sample and had hair metal concentrations that were higher than cluster 1 but still fairly low. Six percent of the sample was represented by the third cluster (“high lead and cadmium”), which had mean hair cadmium and blood lead concentrations higher than the previous two clusters. The fourth cluster (2% of the sample) had elevated mean concentrations of arsenic, lead, and manganese, but not cadmium, and was named “high metal exposure.” In terms of mean blood lead levels, cluster 2 (low-to-moderate exposure) had the lowest and cluster 3 (high lead and manganese) had the highest mean concentrations.

No statistically significant associations were found between clusters of metal exposure and any of the cognitive performance scales, either in the complete case or the multiple-imputation analysis (Table 4). There were similarities between the complete-case and multiple-imputation analyses in that differences in scores tended to get larger between the reference group and the clusters representing more “severe” exposure. This is particularly interesting because these differences suggested better performance in cluster 4 than in the low-exposure reference group. However, particularly in the multiple-imputation analysis, the error around the estimates was very large, indicating a high level of uncertainty.

## 4. Discussion

To better protect human health, there is a need to understand the effects of exposure to chemical mixtures [51], including metals. However, because only a handful of studies have examined the potential synergistic effects of multiple metals on neurodevelopmental outcomes in children, there is little existing evidence on these relationships. We used LCA to describe patterns of exposure to multiple metals (arsenic, cadmium, manganese, and lead) in young children living in Uruguay. We found no associations between children's exposure to multiple metals and their performance on cognitive and language scales of the Bayley Scales of Infant Development III.

There are several possible explanations for these findings, as discussed below. First, it could be that low-level environmental exposure, even to multiple metals, does not produce neurocognitive deficits in young children. Given the epidemiological and animal studies published to date, this explanation is not very likely. Several animal studies have shown interactions among toxic metals on the accumulation of metals in different brain regions [18], neurotransmitter metabolism [19, 20], and activity [21]. Thus, it is reasonable to suspect that metal interactions would affect several cognitive functions in humans. Furthermore, because many of the metals in question appear to act in similar ways (the dopaminergic system is one major target of toxicity and all contribute to oxidative stress), there is a reasonable expectation that together they would produce multiplicative effects even at low doses, the concept of concentration addition [52].

In a recent review, Claus Henn et al. [51] concluded that the toxicity of lead appeared to increase when children are coexposed with other metals, such as manganese, arsenic, mercury, and cadmium. Of the studies that have measured at least two metals in children, several have shown evidence of additive or multiplicative effects on cognition or behavior [27, 30–32, 34]. More recently, an interaction was reported between blood lead and cadmium concentrations measured in late pregnancy on children's MDI scores [53]. Similarly, Lin and colleagues [54] found poorer cognitive and language scores in 2-year old children whose mothers had elevated lead and manganese concentrations in cord blood, while Claus Henn and colleagues [32] found synergistic lead-by-manganese effects on Bayley scores in Mexican children.

A second explanation for the null findings in this study is that there was insufficient statistical power to detect the associations. Post hoc power analysis for the final model comparing the four metal exposure groups for a significance level of 0.05, power of 0.80, and a moderate effect size, *f* = 0.25, resulted in an estimated sample size of 44 per group. We did not have an overall sample of this size and only the low-metal exposure cluster included at least 44 participants. Estimating post hoc power for a large effect size resulted in a sample size of 18 per group. The low-metal and low-to-moderate metal exposure clusters met this sample size criterion. Although at an imputed sample size of 92 our study is larger than some previous reports of metal interactions [30], future studies will need to include larger samples to test the neurodevelopmental effects of multiple-metal exposures. This is particularly important at low and very low level exposures, which may produce small effects.

Third, we used latent class analysis to separate children into metal exposure clusters. LCA is a widely accepted and used statistical approach [48], although its application to environmental exposure is less common. Previous approaches used to evaluate the association between multiple metal exposures and cognitive abilities or performance entered each biomarker separately into regression models to test either the main effects or interactions of the metals, typically in pairs. This study is the first to use LCA to construct independent variables representing metal exposures in children. However, LCA is also somewhat arbitrary in that, being data-driven, it relies on the observed distribution of each metal to classify individuals into groups. As such, depending on the range of exposures in a given population, the mean metal concentratins within clusters may differ among studies. Furthermore, it is also likely that the classes identified and the names given to the groups will differ among studies. These limitations do not detract from the validity of LCA but it is also worth mentioning that other statistical approaches have been used [51].

We identified four patterns of metal exposure using LCA: (1) low-metal exposure, (2) low-to-moderate metal exposure, (3) high lead and cadmium, and (4) high-metal exposure. Several points are worth noting about metal concentrations in these clusters. Mean BLLs fell below 10 *μ*g/dL in all the clusters. While cognitive deficits have been noted in children with low BLLs [36], in this study the high lead and cadmium and high-metal exposure clusters did not appear to differ dramatically on BLL from the reference group and is one potential reason for why no differences in test scores would be seen among these clusters. However, it is important to note that in another study of these children we found no independent associations between blood lead concentrations and Bayley III scores [46]. Furthermore, in our study, mean hair manganese concentrations in clusters 3 and 4 (high lead and cadmium and high-metal exposure, resp.) were above 3 *μ*g/g. Others have reported increased behavior problems in school-age children with hair manganese concentrations above this level [15]. In another study of Uruguayan preschoolers, we found limited evidence of an association between Bayley III scores and hair manganese [38]. On the other hand, Claus Henn and colleagues [55] reported negative associations between blood manganese and Bayley scores at 12 months of age, although there were no associations at later ages. Cadmium exposure has been associated with cognitive deficits in young children [56], but studies are limited and mostly measure blood cadmium [56, 57]. For this reason it is difficult to comment on the range of hair cadmium concentrations in the clusters identified in our study or on their relative contribution to cognitive deficits. This is also true for hair arsenic. To our knowledge, only one other study examined the association of hair arsenic concentrations with children's cognition and found arsenic-by-manganese interactions [30]. Based on this limited evidence, we would have expected but did not find lower cognitive scores in the high-metal exposure cluster compared to the low-metal exposure cluster.

## 5. Conclusions

Using a latent class approach we found no evidence of poorer neurodevelopmental scores in young Uruguayan children exposed to multiple toxic metals. Although LCA has limitations that have implications for cross-study comparison, it also represents an approach that is valid and, importantly, data-driven. Therefore, our study can serve as a model for the use of LCA in environmental exposure science (alone or in combination with multiple imputation to reduce the influence of missing data on point estimates). We recommend that additional studies using the LCA are conducted with larger samples to test for the association with low-level multiple metal exposure and the neurobehavioral development of preschool children.

## Figures and Tables

**Table 1:** Participant characteristics.

Characteristic	*N*	M ± SD or %	Range
Child characteristics			
Age (months)	95	29.1 ± 8.3	13–42
Sex (female)	94	47.9	
Child lives with both parents	72	76.6	
Blood lead (*µ*g/dL)	78	5.8 ± 2.9	2.4–15.5
Hemoglobin (g/dL)	87	12.2 ± 1.6	7.7–16.1
Hair arsenic (*µ*g/g)	86	0.2 ± 1.0	0.01–9.6
Hair cadmium (*µ*g/g)	86	0.2 ± 0.2	0.01–0.9
Hair manganese (*µ*g/g)	86	1.0 ± 1.4	0.2–11.9
BAYLEY cognitive score (raw)	71	62.4 ± 10.7	33–79
Maternal characteristics			
Age (years)	92	28.3 ± 7.1	16–43
Education (years)	92	8.9 ± 3.2	1–19
IQ score	74	81.3 ± 14.6	29–121
Depressive symptom score	86	15.5 ± 10.8	0–52
>19		31.4%	
Employed outside home	90	43.3%	
Family/household characteristics			
Parents separated/divorced	94	21.3%	
Socioeconomic status score	93	6.1 ± 2.4	0–11
Occupant density	91	1.9 ± 1.3	0.6–7
HOME Inventory score	85	8.9 ± 2.4	0–14

**Table 2:** Model comparison for 3 and 4 class cluster models in the latent class analysis.

	3-class model	4-class model
Log-likelihood (LL)	−133.7084	−109.025
BIC (based on LL)^1^	389.3919	382.2474
AIC (based on LL)^2^	319.4169	288.0502
CAIC (based on LL)^3^	415.3919	417.2474

^1^Bayesian Information Criterion; ^2^Akaike Information Criterion; ^3^Corrected Akaike Information Criterion.

**Table 3:** Mean hair arsenic, cadmium, manganese, and blood lead concentrations in participant groups according to the latent class analysis.

	Cluster 1 “Low metal exposure”	Cluster 2 “Low-to-moderate metal exposure”	Cluster 3 “High lead and cadmium”	Cluster 4 “High metal exposure”
*Cluster size^1^*	*0.7231 *	*0.1909 *	*0.0636 *	*0.0224 *
Hair manganese (*µ*g/g)^2^	0.7081	1.2271	3.2436	**6.5836**
Hair arsenic (*µ*g/g)^2^	0.0726	0.1733	0.3878	**5.6398**
Hair cadmium (*µ*g/g)^2^	0.0973	0.2884	**0.6294**	0.1222
Blood lead (*µ*g/L)^2^	6.4138	4.4624	**8.3307**	7.4579

^1^Values given as proportion of the study sample represented by the cluster; ^2^values given as mean concentration of a given metal in each cluster; highest mean concentrations of each metal appearing in bold.

**Table 4:** Associations between multiple-metal exposure and raw developmental scores on the Bayley Scales of Infant Development III.

Outcome	Cluster 2 “Low-to-moderate metal exposure” *β* ± SE	Cluster 3 “High lead and cadmium” *β* ± SE	Cluster 4 “High metal exposure” *β* ± SE
Complete-case analysis^1,2,3^			
Cognitive scale	−1.42 ± 1.95	−1.27 ± 2.76	1.64 ± 3.83
Expressive vocabulary scale	−1.13 ± 2.53	0.48 ± 3.58	3.96 ± 4.97
Receptive vocabulary scale	−0.32 ± 2.33	2.28 ± 3.30	6.70 ± 4.56
Imputed-data analysis^1,2,3^			
Cognitive scale	0.35 ± 2.68	1.72 ± 3.35	5.10 ± 16.23
Expressive vocabulary scale	2.24 ± 4.89	4.54 ± 4.95	0.59 ± 24.08
Receptive vocabulary scale	1.34 ± 4.04	3.87 ± 3.91	12.07 ± 25.85

^1^Sample size was 47–49 for complete-case analysis and 92 for models with imputed data; ^2^cluster 1 (“Low metal exposure”) served as the reference group; ^3^models adjusted for child age in months, maternal IQ and depressive scores, household density, HOME score, socioeconomic status, tester, and the child's hemoglobin value.
